# Fundamental aspects of electric double layer force-distance measurements at liquid-solid interfaces using atomic force microscopy

**DOI:** 10.1038/srep32389

**Published:** 2016-09-02

**Authors:** Jennifer M. Black, Mengyang Zhu, Pengfei Zhang, Raymond R. Unocic, Daqiang Guo, M. Baris Okatan, Sheng Dai, Peter T. Cummings, Sergei V. Kalinin, Guang Feng, Nina Balke

**Affiliations:** 1 Center for Nanophase Materials Sciences, Oak Ridge National Laboratory, Oak Ridge, TN, 37831, USA; 2 State Key Laboratory of Coal Combustion, School of Energy and Power Engineering, Huazhong University of Science and Technology (HUST), Wuhan 430074, China; 3 Chemical Sciences Division, Oak Ridge National Laboratory, Oak Ridge, TN, 37831, USA; 4 Department of Chemical &Biomolecular Engineering and Multiscale Modeling and Simulation Center, Vanderbilt University, Nashville, TN, 37235, USA

## Abstract

Atomic force microscopy (AFM) force-distance measurements are used to investigate the layered ion structure of Ionic Liquids (ILs) at the mica surface. The effects of various tip properties on the measured force profiles are examined and reveal that the measured ion position is independent of tip properties, while the tip radius affects the forces required to break through the ion layers as well as the adhesion force. Force data is collected for different ILs and directly compared with interfacial ion density profiles predicted by molecular dynamics. Through this comparison it is concluded that AFM force measurements are sensitive to the position of the ion with the larger volume and mass, suggesting that ion selectivity in force-distance measurements are related to excluded volume effects and not to electrostatic or chemical interactions between ions and AFM tip. The comparison also revealed that at distances greater than 1 nm the system maintains overall electroneutrality between the AFM tip and sample, while at smaller distances other forces (e.g., van der waals interactions) dominate and electroneutrality is no longer maintained.

ILs have a number of beneficial properties with applications in many areas including energy storage, catalysis, and lubrication^1^,^2^,^3^,^4^,^5^. In all of these applications the interfacial structure of ILs at the solid-liquid interface plays a crucial role in determining their performance, and therefore it is essential to have techniques able to characterize the interface and build structure-function relationships. In recent years numerous efforts have gone toward understanding the complex structure of ILs at the solid-liquid interface using both theoretical approaches^6^,^7^,^8^,^9^,^10^,^11^,^12^ and experimental methods such as scattering techniques^13^,^14^,^15^, sum-frequency generation^16^,^17^,^18^, surface-force apparatus^19^,^20^,^21^,^22^,^23^,^24^,^25^, and scanning probe techniques. For the latter, scanning tunneling microscopy (STM)^26^,^27^,^28^,^29^,^30^,^31^,^32^,^33^,^34^,^35^,^36^ and dynamic^37^,^38^,^39^,^40^,^41^ and static^42^,^43^,^44^,^45^,^46^,^47^,^48^,^49^,^50^,^51^ atomic force microscopy (AFM) approaches have made large progress towards imaging the ion layers in two and three dimensions at neutral and charged surfaces such as mica, silica, gold, and highly-oriented pyrolytic graphite (HOPG). Scanning probe microscopy based techniques offer the advantage of high spatial resolution in three dimensions compared to other techniques, allowing for the ion structure to be visualized in a 3D manner, as opposed scattering techniques and SFA where the response is averaged over large areas.

Although there are numerous publications using AFM techniques to study the IL-solid interface, the signal generating mechanism is still not fully understood. Specifically, this includes the role of the various AFM tip properties and the ion selectivity, which determines which ion of the ionic liquid the probe will detect. We showed in a previous publication for the IL Emim^+^ Tf_2_N^−^at an HOPG substrate that the positions of the ion layers measured by AFM force-distance measurements coincided with the position of the anion, Tf_2_N^−^, predicted by molecular dynamics (MD) simulation^45^. The reason for this selectivity to the anion is not well understood, and it raised the question of whether we can tune the system to be sensitive to either the cation or anion by either changing properties of the AFM probe or the ionic liquid. The aim of this work is to discuss how various properties of the AFM probe affect the measured force response, such as stiffness, tip material, tip geometry and cleaning procedures, as well as to identify the origin of the selectivity of the force measurements to a particular ion of the ionic liquid. To do this, we performed static force-distance measurements on freshly cleaved mica substrates in several different ILs using a range of AFM probes with differing properties, and compared results with the mica-IL interfacial structure obtained by MD simulation.

## Methods and Materials

### Experimental

Static force-distance curves were collected on a Cypher AFM from Asylum Research (Santa Barbara, CA) under ambient conditions in a droplet of room temperature ionic liquid on a freshly cleaved mica surface. A minimum of 50 curves were collected at a rate of 0.5 Hz and 2D histograms of the measured curves were constructed to achieve statistical significance. Three different types of AFM probes were used for the measurements having cantilevers of equal geometry: uncoated silicon nitride tip with a spring constant of ca. 0.6 N/m; Au-coated silicon nitride tip with a spring constant of 0.6 N/m; and Au-coated silicon nitride tipless cantilever with a spring constant of 0.24 N/m. All tips were calibrated using the thermal tune method. Tips were either used as delivered or cleaned prior to measurements where indicated by placing the tip in a UV/Ozone cleaner (Surfinator Pro, Creodyne LLC) for 5 minutes. In order to compare all curves and account for drift, the curves were aligned along the y-axis to be zero force far away from the sample, and aligned along the x-axis so that the retract branch of all force-separation curves overlaid. The tip-sample separation was calculated by subtracting the cantilever deflection from the z piezo position. The radii of AFM tips were determined from AFM and scanning transmission electron microscopy (STEM) images. AFM images were collected by scanning the AFM tip in contact mode over a TGT1 test grating (NT-MDT, Russia) containing an array of sharp tips with a radius <10 nm. Tip radii were calculated from AFM images using the tip characterization tool in the Scanning Probe Image Processor (SPIP) software. Tips were imaged as received prior to any force measurements as well as after being subjected to several hundred force-distance measurements. AFM tips were also directly imaged using a Hitachi HF3300 S/TEM instrument, operating at 300 kV. The AFM tips were mounted onto a specialized *in situ* TEM holder and images of the tip were acquired in bright-field scanning transmission electron microscopy (BF-STEM) imaging mode. The radius of the tip was measured using quantitative image analysis techniques.

The imidazolium ILs 1-ethyl-3-methylimidazolium bis(trifluoromethylsulfonyl)imide (Emim^+^Tf_2_N^−^), 1-ethyl-3-methylimidazolium tetrafluoroborate (Emim^+^BF_4_^−^), and 1-butyl-3-methylimidazolium hexafluorophosphate (Bmim^+^PF_6_^−^) were synthesized according to standard procedures^52^,^53^. For example, Emim^+^Tf_2_N^−^ was synthesized from the reaction of 1-ethyl-3-methylimidazolium chloride (Emim^+^Cl^−^) with lithium bis(trifluoromethylsulfonyl)imide (Li^+^Tf_2_N^−^) in water and dried at 100 °C under vacuum. Before use, all the ILs were freeze-dried for three days, to keep the water contents lower than 100 ppm (as measured by Karl Fischer titration).

### Simulation

The MD channel system consists of a slab of ILs enclosed between two mica walls, and each wall was modeled as two mica layers, with a surface area of 4.243 × 4.59 nm^2^ and thickness of 2.04 nm^54^. The planes through innermost oxygen atoms on the mica surface was taken as 0 nm to gauge the location of ILs towards mica walls; the distance between such planes of two mica walls is set as 8.0 nm, which is large enough to ensure the bulk-like ILs in the central portion between two mica walls. The force fields for mica atoms were taken from ref. 54. The number of K^+^ ions on the mica surface could be tuned to have a mica wall with different surface charge densities due to the water rinse, and in this work the mica surface was modeled with 50% K^+^ ions left. The amount of cations/anions between mica walls was tuned to guarantee the neutrality of the system and bulk state of ILs in the center of the channel.

To investigate the size effects on interfacial IL structure, ILs Emim^+^Tf_2_N^−^, Emim^+^ BF_4_^−^ and Bmim^+^ PF_6_^−^ were used in MD simulation with corresponding all-atom models and force fields developed by Lopes group^55^,^56^. Simulations were performed in the NVT ensemble using a customized version of the MD code Gromacs^57^. The electrolyte temperature was maintained at 298 K using the Nosé-Hoover thermostat. The time step of 2 fs was used to integrate the equations of motion with spherical cutoff of 1.1 nm in non-bonded van der Waals interactions. To compute the electrostatic interactions in the two-dimensional periodic geometry adopted here, the slab-PME method was used. The dimension of the simulation box in the channel width direction was set to be 4.5 times the channel width^58^. An FFT grid spacing of 0.1 nm and cubic interpolation for charge distribution were used to compute the electrostatic interactions in the reciprocal space. A cutoff length of 1.1 nm was used in the calculation of electrostatic interactions in the real space. For each simulation, the MD system was first simulated at 1000 K for 3 ns, and the system temperature was gradually quenched to the target temperature. After an equilibration of 30 ns, a 120 ns production run was generated for data analysis.

## Results and Discussion

ILs generally form ordered layered structures at solid interfaces consisting of alternating layers of cations and anions^59^,^60^,^61^. This interfacial structure can be probed using atomic force microscopy (AFM) force-distance measurements. As the AFM tip slowly moves through the ionic liquid toward the substrate and encounters an ion layer, there is a resistance inhibiting the AFM tip from penetrating the ion layer. This causes the cantilever to deflect until enough force is applied to break through the layer, and the tip moves closer to the surface to the next ion layer. The amount of force required to break through the ion layers increases exponentially as we get closer to the surface, as the ion layers become denser and more ordered. After reaching a predetermined force set-point the AFM tip is then retracted away from the surface. Force-distance curves are converted into force-separation plots as described in a previous publication^45^. One difficulty with performing AFM force-distance measurements is that the exact tip-sample separation value is not known, and therefore there is uncertainty as to when the tip is truly in contact with the surface.

**Figure 1:**
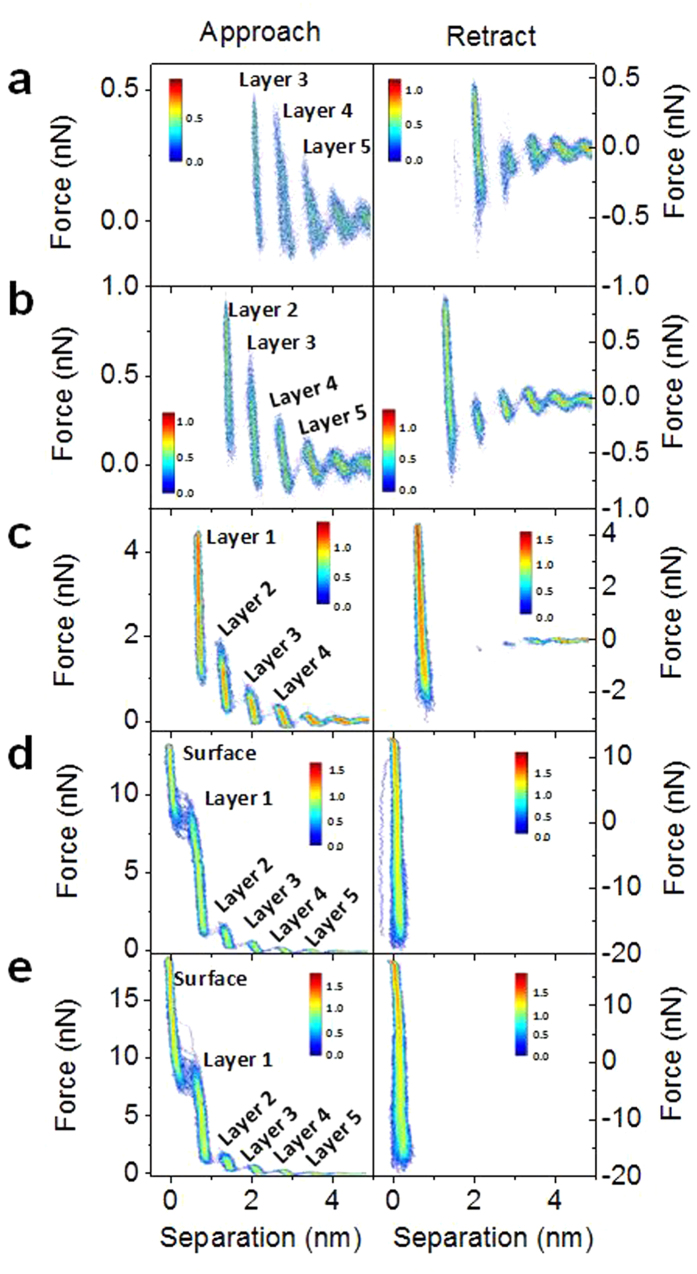
Bivariate histograms of approach (left) and retract (right) portion of 50 measured force-curves in Emim^+^ Tf_2_N^−^ ionic liquid on mica with increasing force set-points as 500 pN (**a**) 1 nN (**b**) 2 nN (**c**) 14 nN (**d**) and 18 nN (**e**) The color bar represents the common logarithm of the frequency of data points in each bin.

To determine when the tip reaches the surface during force-distance measurements, a series of force-measurements with increasing force set-point are performed. Figure 1 shows a series of 2D histograms of force-separation plots each constructed from the approach and retract section of 50 individual force-distance curves in Emim^+^ Tf_2_N^−^ ionic liquid on a freshly-cleaved mica substrate. In Fig. 1a the force set-point is ca. 500 pN, and multiple distinct segments are observed in both the approach and retract segment of the force profile, each segment being associated with an ion layer. At a set-point of ca. 1 nN (Fig. 1b) the tip now penetrates an additional ion layer. When the force set-point is further increased to ca. 4.5 nN (Fig. 1c) the tip penetrates yet another ion layer. At this set-point the tip starts to jump through one or two ion layers during retraction and only shows a few ion layers further away from the surface. In addition, the adhesion force (minimum force observed during retract portion of force-distance measurement) increases slightly from ca. 1 nN to ca. 2 nN. At a set-point of ca. 14 nN (Fig. 1d) the tip penetrates through an additional ion layer and the adhesion force increases drastically to ca. 20 nN. In addition to the increase in adhesion force, there are no longer any ion layers observed during the retract portion of the force measurements since the tip snaps off the surface at high force and through the ion layers. With the fact that no additional ion layers are observed when the set-point is further increased to 18 nN (Fig. 1e), and a drastic change in the adhesion force is observed at set-points of >14 nN (Fig. 1c–e), we conclude that at forces >10 nN the AFM tip is in contact with the mica surface. It is important to note that the force required to reach the surface will vary strongly for different AFM tips and this will be discussed in more detail below. Therefore, in our experiments, it is determined that the tip contacts the surface (separation of 0 nm is assigned to the surface) when no additional ion layers are observed with increasing the force set-point (>30 nN) coupled with an increase in the adhesion force (see also Fig. S1 in the Supplementary Material). All of the data in Fig. 1a–e is plotted vs the tip-sample separation value determined as described above. After ensuring the tip has reached the surface at high set-points, we are able to assign the individual steps in the various force curves to the different ion layers as determined by the force required to break through the ion layer which does not change for different experimental set-points. At set-point forces of 14 and 18 nN (Fig. 1d,e) the first line corresponds to the surface and the following peaks to the first, second, third ion layer and so on. At a set-point of 4.5 nN the tip was unable to penetrate through the last ion layer, and therefore the first peak corresponds to the first ion layer rather than the surface, which corresponds to an actual tip-sample separation of ca. 0.7 nm. At a set-point of 1 nN the tip is not able to penetrate the first two ion layers, and therefore the first peak corresponds to the second ion layer, and the actual tip-sample separation of ca. 1.4 nm. And finally at a set-point of 500 pN the tip is not able to penetrate through the first 3 ion layers, meaning the first peak corresponds to layer 3 with a true tip-sample separation value of ca. 2.1 nm.

**Figure 2:**
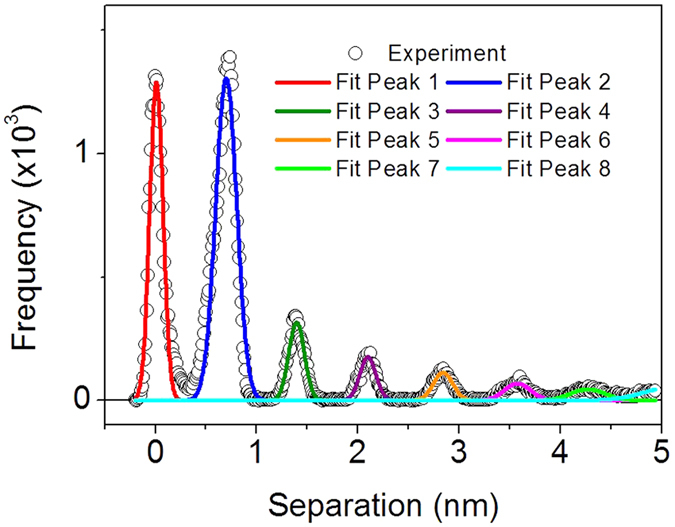
Histogram of separation values for data shown in Fig. 1d and multiple Gaussian functions used to fit experimental data.

To allow for an easy comparison of force curves collected using different AFM probes and for different ILs or substrates, the 2D force-separation histograms can be simplified as histograms of the separation values, as shown in a previous publication^44^. Figure 2 shows a histogram of the separation values for the data shown in Fig. 1d, which was constructed from the approach portion of 50 consecutively-measured force curves in Emim^+^ Tf_2_N^−^ ionic liquid on a freshly cleaved mica substrate. Several peaks are observed in the separation histogram corresponding to each ion layer and the substrate (at 0 nm separation). The separation histogram can easily be fit using several Gaussian functions, with the peak positions providing an accurate measure of the ion layer positions, and the peak width correlating to the degree of ordering within the ion layer, sharper peaks being associated with a higher degree of order as introduced in ref. 44.

**Figure 3:**
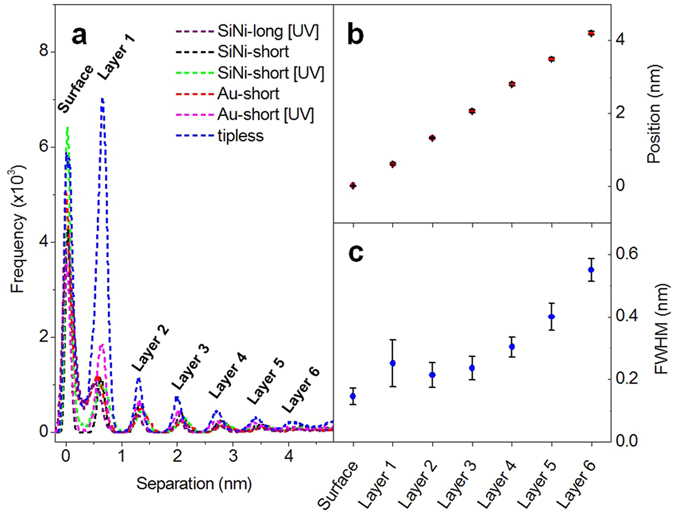
(**a**) Separation histograms for 50 force-curves in Emim^+^ Tf_2_N^−^ ionic liquid on mica for six different AFM probes. Average peak positions (**b**) and full width at half maximum (**c**) obtained by fitting separation histograms shown on the left with Gaussian functions. Error bars represent one standard deviation.

To examine the effect of tip properties on the measured force curves, force measurements were performed in Emim^+^ Tf_2_N^−^ ionic liquid on a freshly-cleaved mica surface using six different AFM probes. The first probe is a silicon nitride (SiNi) tip with a spring constant of 0.20 N/m and a length of 200 μm, which was cleaned in a UV-ozone cleaner prior to measurements (SiNi-long [UV]), second and third are SiNi tips with k = 0.66 N/m and a length of 100 μm without and with ozone cleaning (SiNi-short and SiNi-short [UV]), respectively, fourth and fifth are Au-coated SiNi tips with a length of 100 μm without ozone cleaning (k = 0.67 N/m, Au-short) and with ozone cleaning (k = 0.63 N/m, Au-short [UV]), and finally a Au-coated SiNi tipless cantilever with a length of 200 μm, k = 0.24 N/m (tipless). This group of probes contain cantilevers with different spring constants (ranging from 0.2 to 0.67 N/m), tips of different surface material (SiNi or Au), different geometry (with or without tips) and tips which have or have not been cleaned prior to measurements in a UV-ozone cleaner. This allows us to examine the effect of various tip properties (tip stiffness, tip geometry, and tip chemistry) on the measured force curves. Figure 3a shows the separation histograms for the force curves collected using the 6 AFM probes in Emim^+^ Tf_2_N^−^ ionic liquid on a freshly cleaved mica substrate. Figure 3b,c show the average peak position and full width at half maximum (FWHM) obtained from fitting of the separation histograms as shown in Fig. 2b, and the error bars correspond to one standard deviation. As evident in Fig. 3b, the position of the peak is independent on the properties of the AFM probe. The FWHM of the peaks (Fig. 3c) shows a larger spread, with the largest error associated with peak 2, the first ion layer adjacent to the surface, and the smallest for peak 1, the mica surface. There is an overall trend of increasing peak width with increasing separation from the surface, which is related to the increasing disorder in the system as we move from the highly ordered layers closest to the surface to the disordered bulk phase.

**Figure 4:**
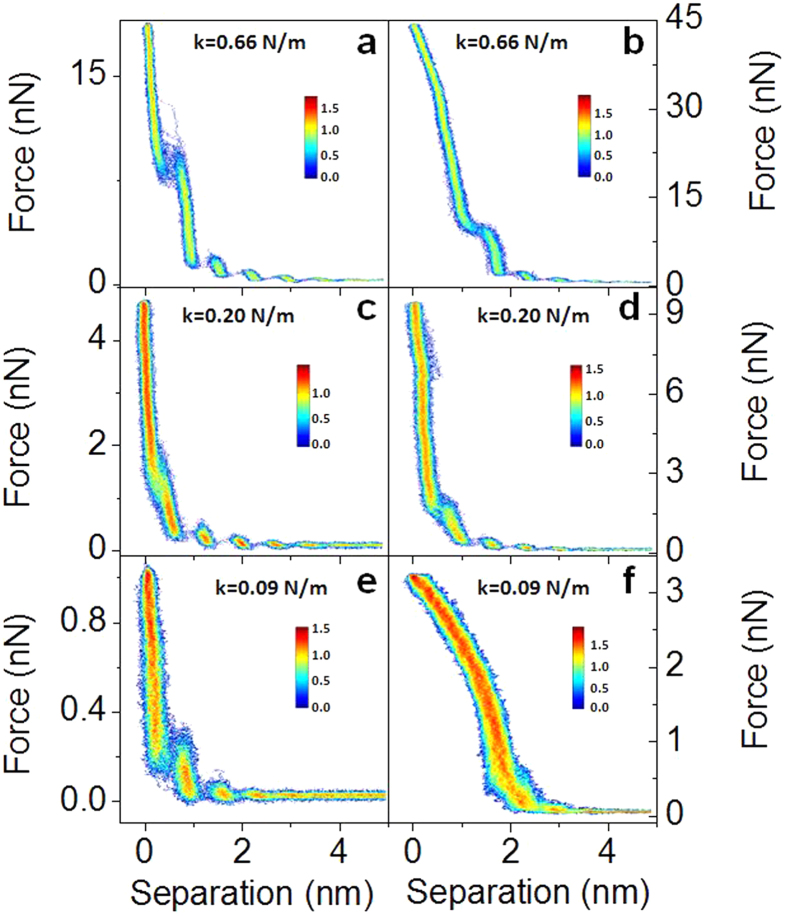
2D histograms of constructed from the approach section of 50 consecutively measured force-distance curves collected with AFM probes having a spring constant of 0.66 N/m (**a**,**b**), 0.20 N/m (**c**,**d**) and 0.09 N/m (**e**,**f**).

As shown in Fig. 3, the stiffness of the AFM cantilever does not affect the measured position of the ion layers for spring constants between ca. 0.2 and 0.67 nN. However, the spring constant is an important parameter when choosing an AFM probe to perform these types of measurements. Cantilevers that are too stiff will not deflect when encountering an ion layer, and therefore softer cantilevers are ideal for these measurements due to their higher sensitivity to weak forces. However, if the cantilever is too soft, it may not be able to puncture through the ion layers to reach the surface before reaching the maximum detectable cantilever displacement as determined by the AFM photodetector. Figure 4 shows 2D histograms of 50 force curves in Emim^+^ Tf_2_N^−^ ionic liquid on mica collected using AFM probes with different spring constants (0.66, 0.20, and 0.09 N/m) in low and high force regimes. Figure 4a,b show data collected using a cantilever with a spring constant of 0.66 N/m. As discussed previously, in order to determine whether the AFM tip contacts the surface, force measurements are collected with increasing set-point forces until no additional ion layers are observed. However, as the force increases from 18 nN (Fig. 4a) to values of 30 nN and above the force-curves become distorted due to non-linearity in the photodetector (Fig. 4b) The force at which this distortion will start to appear will decrease as the stiffness of the cantilever decreases. Figure 4c,d show data collected using a cantilever with a spring constant of 0.20 N/m. Up to 5 nN the system shows a linear response, and in this case the AFM tip is able to contact the surface, but the response becomes non-linear at forces > ca. 7 nN, much lower than of the cantilever with the spring constant of 0.66 N/m (Fig. 4a,b). For even lower spring constants (Fig. 4e,f) the response becomes non-linear at even smaller forces, in this case > ca. 1 nN for a spring constant of 0.09 N/m. Here the tip was unable to penetrate all of the ion layers to reach the surface within the linear range of the photodetector (compare the maximum force in Fig. 4f with the forces to break through ion layers in Fig. 1e).

**Figure 5:**
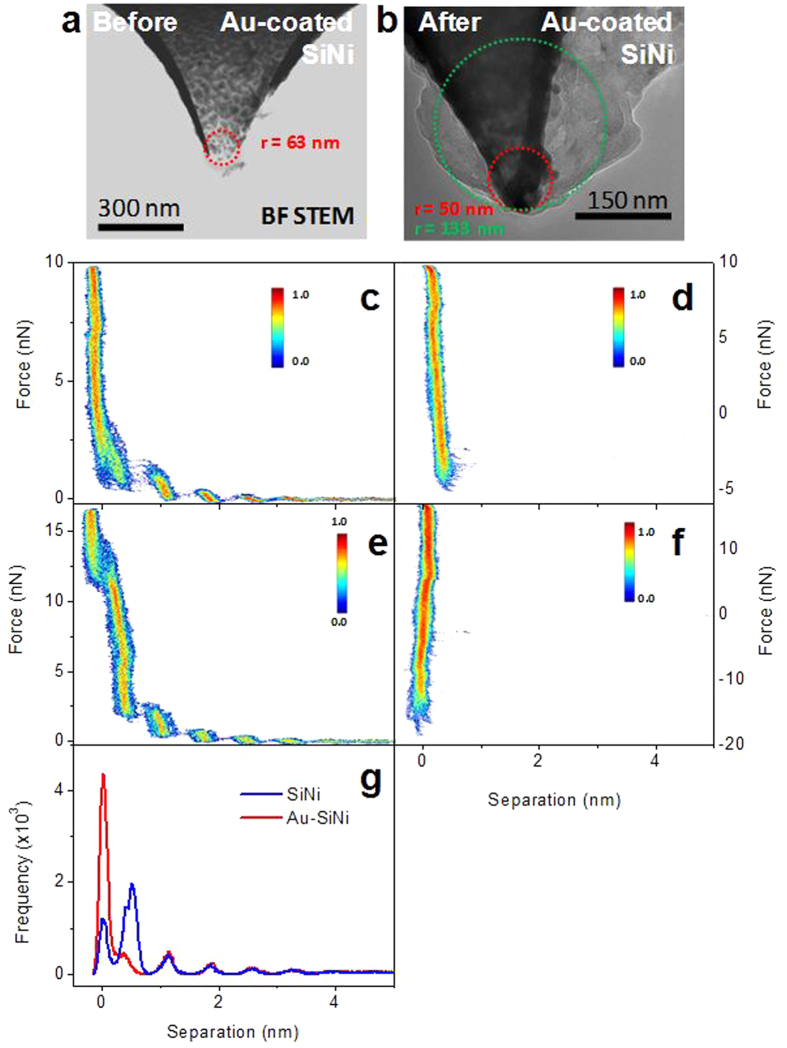
Bright field STEM image of a pristine Au coated SiNi tip (**a**) and one after the performance of several hundred force-distance measurements (**b**). 2D histogram for the approach (**c**,**e**) and retract (**d**,**f**) portion of 50 force-curves in Emim^+^ Tf_2_N^−^ ionic liquid on mica for the SiNi (**c**,**d**) and Au- coated SiNi (**e**,**f**) tips. (**g**) Separation histograms of data shown in panel (**c**,**e**).

It was previously shown theoretically that the radius of the AFM tip will directly affect the forces required to break through the ion layers without affecting the position or number of measured ion layers^62^. Since the actual radius of the AFM tips will vary from tip to tip and from the nominal radius provided in manufacturers specifications, characterization of a couple of AFM probes (an uncoated SiNi tip and a Au-coated SiNi tip) was carried out to accurately determine the tip radius and its effect on measured force-curves. To determine the tip radius, tips were scanned over a test grating containing ultra-sharp tips providing a 3D visualization of the scanning tip. Images of the tip were collected prior to, as well as following the force-measurements. The tip radius was determined from these images using the tip characterization tool in SPIP software. The tip radii for the SiNi tip were determined to be 47 nm (before force measurements) and 42 nm (after force measurements), and therefore little change is observed in the radius before and after measurements. For the Au-coated SiNi tip the calculated tip radii were 66 nm (before force measurements) and 137 nm (after force measurements), indicating a significant increase in the tip radius. Figure 5a,b show scanning transmission electron microscopy (STEM) images of a pristine Au-coated SiNi probe and a Au-coated SiNi probe after being subjected to several hundred force-distance measurements. The radius of the pristine AFM tip determined from the STEM image was 63 nm. The STEM image of the Au-coated SiNi probe taken after the force measurements shows the presence of additional material at the tip apex, likely from the tip picking up contamination or due to wear on the tip coating. Without taking this into account, the tip radius is estimated to be 50 nm from the STEM image. However, it is possible that this material on the AFM probe is stable and has effectively increased the AFM tip radius to ca. 133 nm, as estimated from the STEM image. This would match closely with the radius value obtained from AFM images. Figure 5c,d show the 2D histogram of the approach and retract portion of force curves collected with the SiNi probe, and Fig. 5e,f show the 2D histogram of the approach and retract portion of force curves collected with the Au-coated SiNi tip. The separation histograms for the attract portion for both the SiNi and Au-coated SiNi probes are shown in Fig. 5g. The force required to break through the first layer adjacent to the surface is ca. 2 nN for the SiNi tip (0.31 mPa) and ca. 12 nN (0.20 mPa) for the Au-coated SiNi tip. The adhesion force from the retract portion of the curves is ca. 4 nN (0.63 mPa) for the SiNi tip and ca. 15 nN (0.26 mPa) for the Au-coated SiNi tip. It is clear from the separation histograms (Fig. 5g) that the position of the measured ion layers does not depend on tip radius, however the adhesion force and the force required to puncture the ion layers are strongly correlated with tip radius.

Previous work by our group using Emim^+^ Tf_2_N^−^ ionic liquid at an HOPG interface revealed the measured peak positions matched with predicted positions of anion layers from molecular dynamics (MD) simulations^45^. To investigate how the ionic liquid properties affect the measured force curves and ion sensitivity, force curves were collected for several ILs: Emim^+^ Tf_2_N^−^, Emim^+^ BF_4_^−^, and Bmim^+^ PF_6_^−^, on freshly-cleaved mica, using an uncoated SiNi tip. Table 1 lists the mass, volume, and dimensions of all of the ions used in this study.

**Figure 6:**
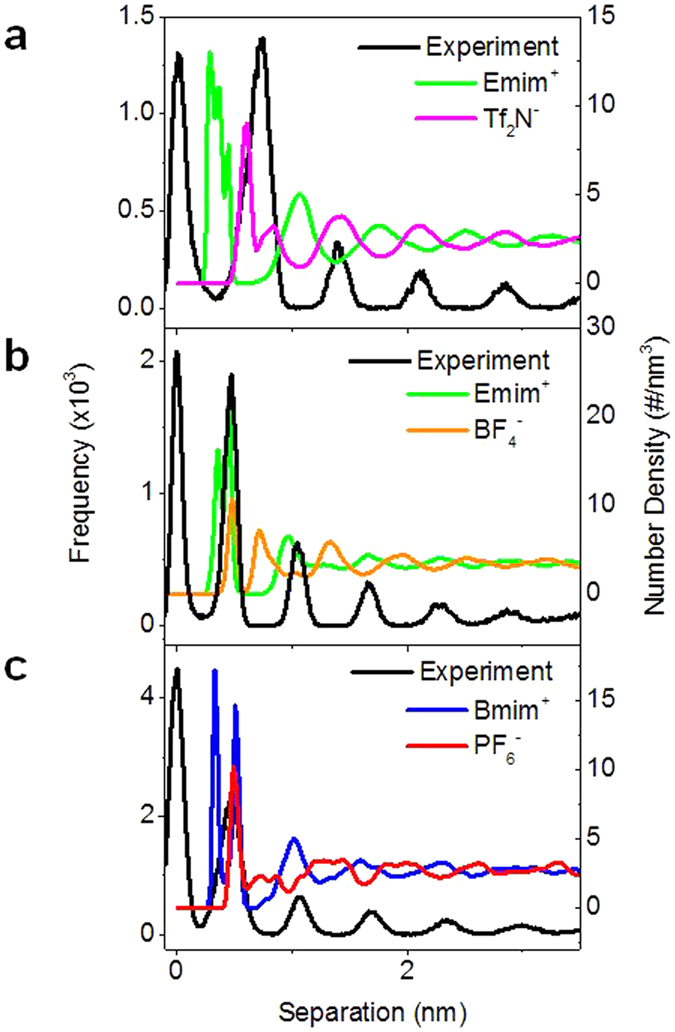
Separation histograms and MD-obtained ion number density profiles for Emim^+^ Tf_2_N^−^ (**a**) Emim^+^ BF_4_^−^ (**b**) and Bmim^+^ PF_6_^−^ (**c**) on mica surfaces.

Figure 6 shows the separation histograms for each of the ILs along with the number density profiles of the cations and anions calculated from MD simulation, based on the center of mass of each ion. Figure 6a shows the profiles for Emim^+^ Tf_2_N^−^ ionic liquid. The peaks in the separation histogram match very closely with the peaks of the number density profile for the anion, Tf_2_N^−^. This agrees with what was observed previously on an HOPG substrate^45^. This is another indication that we are indeed reaching the surface and displace the ion layer directly absorbed to the surface. If we would assume that there is still one cation layer or even one cation and one anion layer left on the surface, the experimental curves do not match the position of either ion predicted by MD simulations (see also Fig. S2 in the Supplementary Material). Figure 6b shows the profiles for the Emim^+^ BF_4_^−^ ionic liquid. In this case, the peaks in the separation histogram match closely with the peaks in the number density profile for the cation, Emim^+^. The Emim^+^ Tf_2_N^−^ and Emim^+^ BF_4_^−^ ILs contain the same cation, however in the former case we measure the position of the anions, and the position of the cations in the latter. For Emim^+^ Tf_2_N^−^ the mass and volume of the anion are larger than the cation (Table 1), whereas for Emim^+^ BF_4_^−^ the cation has the larger mass and volume. This then suggests that during AFM measurements we are sensitive to the ion which has the larger mass and/or volume. To determine whether mass or volume is the important parameter, we also looked at an ionic liquid which has similar ion mass. For Bmim^+^ PF_6_^−^ the anion has a similar mass but smaller volume, compared with the cation Bmim^+^. Figure 6c shows the separation histogram and number density profiles from MD simulation for the Bmim^+^ PF_6_^−^ ionic liquid. Here, the first peak in the separation histogram matches to the combined positions of the cations and anions predicted by MD, and the other peaks match with cation peaks in MD. These observations suggest the ion volume plays an important role in determining the ion layers probed by AFM tips, and MD-predicted interfacial IL structures also render solid support for the experimental finding that the position of the ion layer is independent on the properties of the AFM probe and the tip size. This finding together with the insensitivity to tip radius or chemistry demonstrates that the ion selectivity in force-distance curve measurements is based on excluded volume and molecular mass effects and not on electrostatic or chemical interactions between ions and AFM tip.

**Figure 7:**
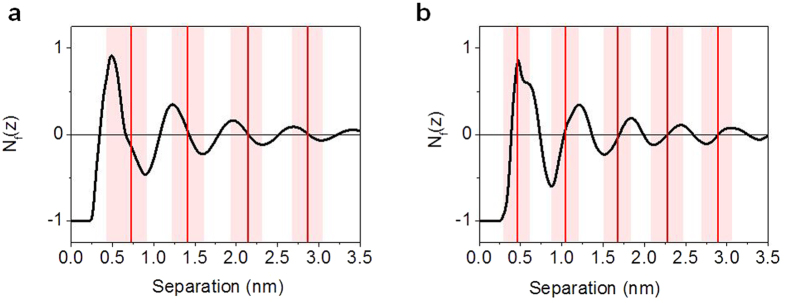
Plot of N_f_ for Emim^+^ Tf_2_N^−^ (**a**) and Emim^+^ BF_4_^−^ (**b**). Red lines indicate the peak positions from experimental data shown in Fig. 6a,b. The red shades represent the range of separation values over which the peak occurs.

Figure 7 shows a plot of the neutral ion number factor (N_f_) calculated from the following equation:


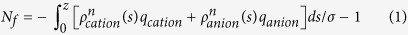


Where ρ is the ion density, q is the ion charge, s is the separation value or distance from the surface and σ is the surface charge of the mica. Figure 7a shows a plot of N_f_ for Emim^+^ Tf_2_N^−^ ionic liquid. The red lines in the figure indicate the peak positions observed experimentally, shown in Fig. 6a and the shaded regions show the range of separation values over which the peak occurs. The onset of the experimental peak corresponds to the minima in the N_f_ curve calculated from MD, and extends to the maxima in the N_f_ curve. Throughout this range of separation values a net number of anions are removed from between the surface and the tip. The experimental peak maxima occur at the zero value of N_f_ (N_f_ = 0 means the ILs between this location and the mica surface could exactly balance the surface charge). This indicates that the ions are removed in such a way as to maintain electroneutrality between the tip and the mica surface. This result is in agreement with previous computational work^63^,^64^. For the first peak closest to the surface, the experimental peak occurs at slightly negative N_f_ value suggesting that electroneutrality is not maintained at this separation value.

Figure 7b shows a plot of N_f_ for Emim^+^ BF_4_^−^ ionic liquid. For this ionic liquid the onset of the experimental peak occurs at the maxima in the N_f_ curve and extends to the minima of the N_f_ curve, and throughout this region a net number of cations is removed. As before the experimental peak positions occur at zero N_f_, indicating that the system maintains electroneutrality between the surface and the AFM tip, except in the case of the first peak which occurs at positive N_f_ value, opposite to what was observed for Emim^+^ Tf_2_N^−^. At larger separation values the system maintains electroneutrality, however at distances less than 1 nm this condition is no longer true, which may be due to the fact that there is only one ion layer (0 ~ 0.65 nm) between the tip and surface and other forces become dominant, such as van der waals interaction between the ions and mica surface.

All measurements were performed in ambient environment and the uptake of water in the ionic liquid droplet and its effect on the ion layering is always a concern. Very recently, two comprehensive studies have been published online in how the water content of the ionic liquid affects the measured ion layering^65^,^66^. In both papers, the water content needs to be considerably high ~ 18000 ppm^65^ or around 50 vol% H_2_O^66^ to affect the layering significantly and to induce time effects^65^. These water contents could only be achieved by actively humidifying the ionic liquid or physically mixing it with water and the cross-over from where water needs to be considered is not explored. Additionally, it was found that exposure to atmosphere did not increase the water content to more than 300–500 ppm for Emim-Tf_2_N starting from 100 ppm right after the drying procedure^65^. The relatively small water uptake was also described in our previous work, where the water content of commercial Emim^+^Tf_2_N^−^ changed from 5.7 ppm to only 6.3 ppm after 24 h^44^. In order to estimate the effect of water on the ion layering on mica, additional MD simulations were performed (see Fig. S3 in the Supplementary Material). It was found that water contents up to 10000 ppm did not affect the ion layer positions significantly. Therefore, we conclude that we do not need to consider the effect of water for this study.

## Conclusions

Atomic force microscopy force-distance measurements have proven to be a valuable tool to study the structure of ILs at the solid-liquid interface. In this work we discussed the effect of various tip properties on the measured force response, and demonstrated that the position of the measured ion layers was independent of tip properties such as stiffness, chemistry, geometry, and cleaning procedure.

By comparing the measured force profiles to ion density profiles from molecular dynamics simulations we were able to determine which ion of the ionic liquid the AFM tips are sensitive to during force measurements, and this is determined by the ion volume. By varying the anion or cation of the ILs we are able to move from a regime where we detect the position of the anions (e.g. Emim^+^ Tf_2_N^−^) to one where we detect the position of the cations (e.g. Emim^+^ BF_4_^−^). Using AFM data alone we cannot identify which ion we are sensitive to, and through a direct comparison of experimental data with molecular dynamics simulation, we are able to gain a better understanding of the mechanisms leading to the measured force profiles, therefore making future interpretation of force-curves at ionic-liquid solid interfaces easier. It was shown that at separation values larger than ca. 1 nm the system will remove ions such that overall electroneutrality between the tip and the surface is maintained, however at distances below this electroneutrality is no longer maintained, as other forces (e.g. van der waals) become dominant.

## Additional Information

**How to cite this article**: Black, J. M. *et al*. Fundamental aspects of electric double layer force-distance measurements at liquid-solid interfaces using atomic force microscopy. *Sci. Rep.* **6**, 32389; doi: 10.1038/srep32389 (2016).

## Supplementary Material

Supplementary Information

## Figures and Tables

**Table 1:**
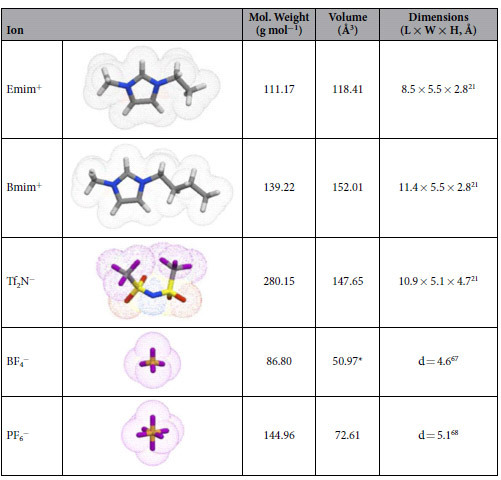
Volume, mass, and dimensions of ions used in this study.

Ion volumes calculated from Molinspiration software. ^*^BF_4_^−^ volume calculated based on sphere of diameter 4.6 Å
