# Supplementary material for: Fundamental aspects of electric double layer force-distance measurements at liquid-solid interfaces using atomic force microscopy
Source: Sci Rep. 2016 Sep 2;6:32389. doi: 10.1038/srep32389 (PMC5009352; doi:10.1038/srep32389)
Supplement: Supplementary Information [file srep32389-s1.pdf]

Supplementary Material

Fundamental aspects of electric double layer force-distance measurements at liquid-solid interfaces using atomic force microscopy

Jennifer M. Black,[1] Mengyang Zhu,[2] Pengfei Zhang,[3] Raymond R. Unocic,[1] Daqiang Guo,[2] M. Baris Okatan,[1] Sheng Dai,[3] Peter T. Cummings,[4] Sergei V. Kalinin,[1] Guang Feng,[2] and Nina Balke[1*]*

[1]Center for Nanophase Materials Sciences, Oak Ridge National Laboratory, Oak Ridge, TN, 37831, USA
[2]State Key Laboratory of Coal Combustion, School of Energy and Power Engineering, Huazhong University of Science and Technology (HUST), Wuhan 430074, China
[3]Chemical Sciences Division, Oak Ridge National Laboratory, Oak Ridge, TN, 37831, USA
[4]Department of Chemical &Biomolecular Engineering and Multiscale Modeling and Simulation Center, Vanderbilt University, Nashville, TN, 37235, USA

[*]Dr. N. Balke (corresponding author experimental – balken@ornl.gov)
[*]Prof. G. Feng (corresponding author theory – gfeng@hust.edu.ch)

The measurement of ion layers using atomic force microscopy (AFM) is widely spread since it can reveal the position of the ion layers in the electric double layer of room temperature ionic liquids and the solid-liquid interface. However, AFM measurements come with the uncertainty (opposite to measurements done by surface force apparatus) that it is not know directly if the AFM tip reaches the surface or if a strongly bound ion layer remains on the sample. In the manuscript, we provide a series of arguments which can be used to gain insight in this question. While none of them alone is enough to conclude that the AFM tip reaches the surface, the combination of all information can be used to reach an answer. This includes:

(i) Increase in penetration force for the ion layer closest to the surface only when the tip radius is increased. The penetration force of other ion layers is independent of tip radius.

(ii) Increase in adhesion force when the tip penetrates the ion layer closest to the surface as a result of tip-sample interactions.

(iii) Agreement between experiment and simulation of ion layer position only under the assumption that the ion layer closest to the surface is penetrated.

In the following, we provide a more detailed discussion for point (ii) and (iii) as well as a discussion of the role of water in the ion layer position. The last one is not only important to AFM-based measurements but for any technique capable of extracting ion layer positions including scattering techniques.

1. Adhesion forces extracted from the FORC force distance curves

From the experimental data shown in Fig. 1, we conclude that forces larger than 10 nN are sufficient to break through the ion layer directly absorbed to the mica surface. One argument for that is the strong increase in adhesion force. We argue that once the ion layer directly at the surface is penetrated, the tip interacts with the surface charges through Coulomb and van der Waals interactions which increase the adhesion force. Technically, this can also happen for a strongly absorbed cation layer on the surface which is not penetrated. In this case, we would expect the interaction forces between the tip and sample to be much lower due to surface charge compensation. The strong increase in adhesion force is therefore interpreted as an indicator of reaching the surface. Below is an image of the measured adhesion force as function of setpoint force as well as the adhesion force as function of assigned layer reached (from Fig. 1 in manuscript). It can be seen that the adhesion force changes linearly with the layer number until we reach the layer denoted with “0”. Here, something drastically changes which we interpret as the tip reaching the surface.

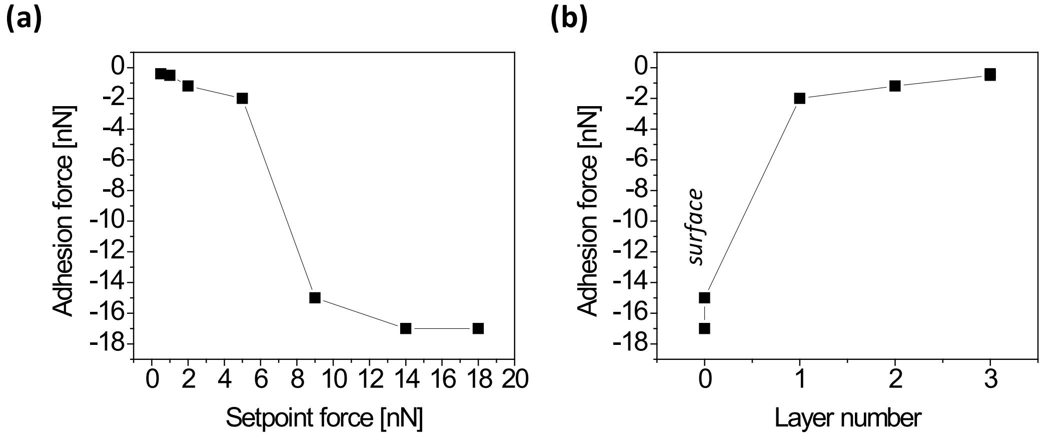

Figure S1. Measured adhesion force as function of setpoint force (a) as well as the adhesion force as function of assigned layer reached (b) extracted from Fig. 1 in the manuscript.

2. Comparison of experimental curve with molecular dynamic simulation

Using force-distance curves based on AFM will always have the uncertainty of whether the surface is reached or not. The strongest argument is that the measured ion positions match the modeling-predicted values with the ion selectivity on top of it. If we would not reach the surface, we can assume that we either have a single ion layer on the surface which we don't penetrate (cation due to the negative surface charge) or even a layer of an ion pair. In this case, we would have to shift the MD-obtained curves with respect to the measured ion position. This is shown below. It can be seen, that only if we assume we penetrate all ion layers, the experiment matches the MD modeling. This shows how important it is to have complimentary MD data to support data interpretation. Therefore, we are confident that we reach the surface and that our assignment of zero separation is correct.

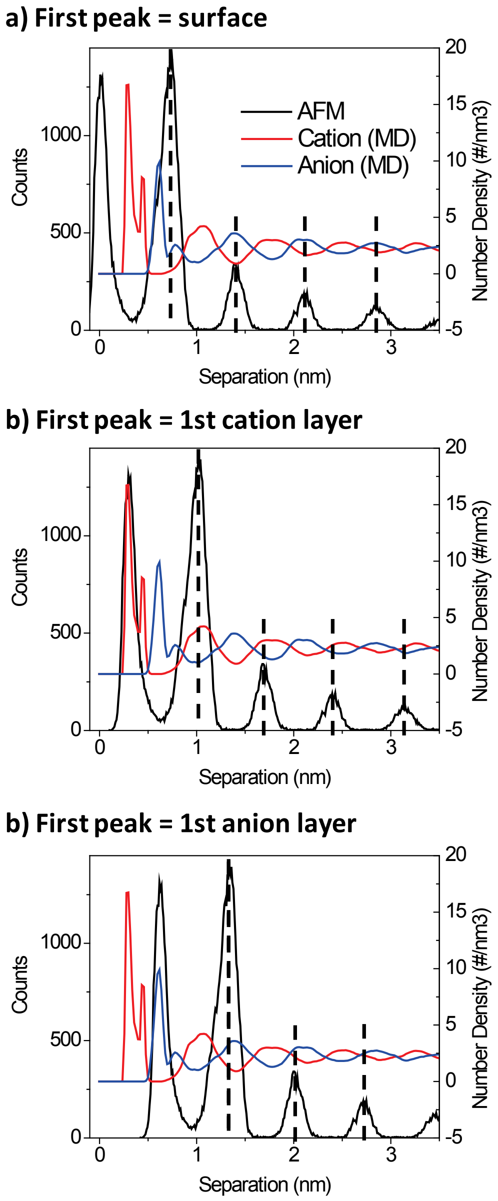

Figure S2. MD curves compared with experimental layer positions if we assume that, (a) we reach the surface, (b) we only reach the cation layer closest to the surface, (c) we only reach the anion layer closest to the surface.

3. The role of water in ion layering on mica

The water content in ionic liquids and the effect on the ion layering is often discussed. We performed MD modeling with different water contents to investigate its affect on ion layering in the case of Emim-Tf_2N on Mica. As can be seen in Fig. S3, the presence of water forms an additional ion layer between the mica (terminated by K^+) and the first cation layer. With increasing water content, the ion number density changes for cation and anions. For example, the width of the 1st cation layer is affected by the water and becomes narrower. However, up to a water content of about 10000 ppm the actual position of the ion

layers as determined by the center of the ion layer peak, remains largely unaltered. Therefore, we conclude that the water content in the ionic liquid is not a concern since water levels this high are difficult to obtain just by performing the experiments in ambient environment.

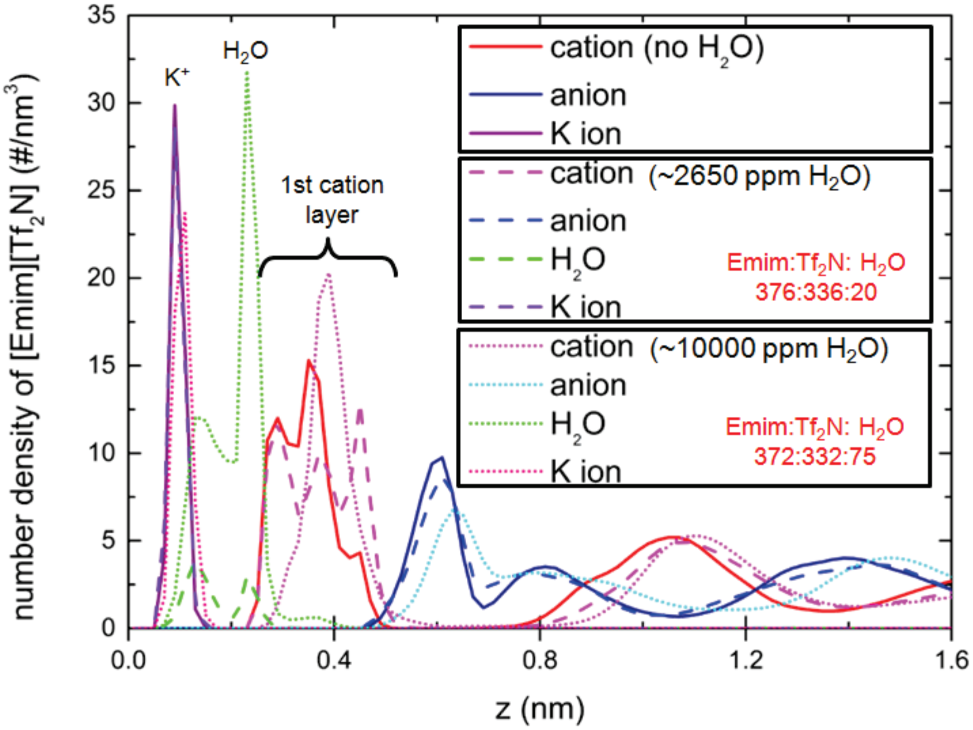

Figure S3. MD simulation of ion density profiles of Emim-Tf_2N on Mica with different water content.
